# Role of Regulated Cell Death Pathways in Snakebite Envenomation: Mechanisms, Crosstalk, and Therapeutic Opportunities

**DOI:** 10.3390/ijms27156830

**Published:** 2026-07-30

**Authors:** Aswathy Alangode, Adithyan Rajasekhar, Jyotsna J. Sabu, Sanjay Krishna, Devika Anuja, Anushree Sreeja, Goutham Remesh, Adithya Kaladevi, Bipin G. Nair

**Affiliations:** School of Biotechnology, Amrita Vishwa Vidyapeetham, Amritapuri, Kollam 690525, India; adithyanrajasekhar786@gmail.com (A.R.); jyotsnajyothy@gmail.com (J.J.S.); aumsanjay04@gmail.com (S.K.); devikanuja02@gmail.com (D.A.); anushreesanilkumar05@gmail.com (A.S.); remeshgoutham@gmail.com (G.R.); adithyaakhil2000@gmail.com (A.K.); bipin@am.amrita.edu (B.G.N.)

**Keywords:** snake venom, snakebite envenomation, regulated cell death, varespladib, apoptosis, ferroptosis, pyroptosis

## Abstract

Snakebite envenomation causes severe tissue damage, often resulting in permanent disability with long-term complications like amputations and organ dysfunction. Current antivenoms, which are antibody-based, have lower tissue penetrability and limited efficacy in minimizing the local effects, highlighting the need for adjunct therapies. Emerging evidence indicates that venom-induced pathology is not restricted to direct cytotoxicity and necrosis; rather, it also involves multiple interconnected Regulated Cell Death (RCD) pathways, but their mechanistic interplay and therapeutic implications remain poorly understood. This review examines how venom toxins induce a cellular stress response characterized by oxidative stress, membrane disruption, and calcium overload, leading to the activation of interconnected regulated cell death (RCD) pathways, including apoptosis, ferroptosis, and pyroptosis, together with autophagy and mitophagy, which primarily function as cellular stress responses that modulate these forms of regulated cell death. We further discuss the crosstalk between these RCD pathways and emerging therapeutic approaches targeting these mechanisms. Understanding these interconnected RCD pathways may facilitate the development of adjunct therapies that complement antivenom, reduce snakebite-induced morbidity, and improve clinical outcomes.

## 1. Introduction

Snakebite envenomation is a serious and neglected public health concern, causing approximately 4.5–5.4 million snakebites and 1.8–2.7 million envenomation cases globally each year. Out of these, about 81,000 to 130,000 deaths occur, with as many as 4,00,000 undergoing amputations and permanent disabilities [1,2,3]. About 1.2 to 2 million envenomation cases, with deaths between 57,000 and 100,000 per year, are reported in Asia [3]. While mortality is a major concern, snakebite envenomation also causes local tissue damage, leading to permanent disability and long-term complications [4]. Tissue damage after a snakebite is primarily driven by venom components like phospholipase A2 (PLA_2_), Snake venom metalloproteases (SVMPs), L-amino acid oxidases (LAAOs) and hyaluronidases [5]. Apart from direct enzymatic effects, there is increasing evidence that points to the involvement of regulated cell death in snakebite-induced tissue destruction, which can cause progressive necrosis, leading to permanent disability and amputations [6]. Current antivenom therapy is effective only in neutralizing venom toxins in the systemic circulation and hence is ineffective in preventing local tissue injury, thus highlighting a critical gap in snakebite management and the need to develop adjunct therapeutic strategies targeting the cytotoxic mechanisms of snake venom toxins [7]. It is now evident that venom-induced tissue damage is not restricted to necrosis but rather involves an interplay between interconnected regulated cell death (RCD) pathways [8]. Understanding the role of host cell death pathways activated by venom toxins in inducing cytotoxicity may open avenues for targeted adjunct therapies to prevent local tissue damage.

Previous reviews have largely focused on venom-induced cytotoxicity, apoptosis, and necrosis, whereas studies implicating other regulated cell death pathways such as ferroptosis and inflammasome-mediated pyroptosis have emerged only recently. Moreover, these RCD pathways are often studied independently, and their mechanistic interplay during snakebite envenomation remains poorly understood. To our knowledge, no review has comprehensively examined the crosstalk between the various RCD pathways and their implications during snakebite envenomation.

Therefore, this review presents an integrated mechanistic framework in which oxidative stress, calcium overload, mitochondrial dysfunction, membrane disruption and inflammation represent common upstream cellular stress responses that act as signaling hubs integrating multiple regulated cell death pathways. This review further discusses how the crosstalk among these RCD pathways contributes to venom-induced tissue damage and highlights these common signaling nodes as a potential therapeutic target for host-directed adjunct therapies that complement conventional antivenom, as well as the limitations and knowledge gaps in the existing studies.

### Literature Search

The literature search was carried out in PubMed, Google Scholar and Science Direct to identify studies on snake venom-induced regulated cell death pathways, including emerging therapeutic interventions. The search included articles published between 2016 and 2026, using keyword search combinations such as: “snake venom and cell death”, “snake venom and apoptosis”, “snake venom and inflammasome”, “snake venom and pyroptosis”, “snake venom and ferroptosis”, “snake venom and autophagy” and “snake venom and small-molecule inhibitors”. Additionally, the literature search primarily focused on studies investigating the effects of snake venom on normal cells and experimental animal models. Studies that exclusively focused on anticancer or antitumor effects of snake venoms in cancer cell lines were excluded as the objective of this review was to evaluate venom-induced toxicity, tissue injury and regulated cell death mechanisms in the context of snakebite envenomation.

## 2. Major Venom Components

Snake venom is a highly complex mixture of pharmacologically active proteins and peptides. Upon envenomation, these toxins act synergistically to trigger severe local tissue damage, inflammation, and life-threatening systemic toxicity. Based on their functional properties and structural domains, these toxins are classified mainly into enzymatic and non-enzymatic protein families.

### 2.1. Enzymatic Venom Components

Snake venom phospholipase A_2_ (svPLA_2_) is one of the most abundant toxin families in snake venom and plays a central role in snakebite envenomation. It hydrolyzes the phospholipids of the cell membrane at the sn-2 position of the glycerophospholipids, thereby releasing free fatty acids and lysophospholipids [9].

Snake venom metalloproteinases are Zn-dependent endopeptidases that are predominantly found in viperid venoms. Based on the presence of the metalloprotease domain, the disintegrin domain, and a cysteine-rich domain, they are categorized into PI, PII, and PIII metalloproteases [10]. SVMPs are the main drivers of hemorrhage, extracellular matrix (ECM), and basement membrane protein degradation [11].

L-amino acid oxidases (LAAOs) are flavoenzymes that catalyze the oxidative deamination of L-amino acids to produce alpha-keto acids and hydrogen peroxide (H_2_O_2_) [12]. The increased release of H_2_O_2_ overwhelms the host’s cellular antioxidant defenses, resulting in severe redox imbalance [13]. As a result, these toxins mediate increased ROS production, which activates intrinsic apoptotic pathways and the NLRP3 inflammasome, thereby inflicting cytotoxicity in keratinocytes, neutrophils, and other tissue cells [14].

### 2.2. Non-Enzymatic Venom Components

Three-finger toxins (3FTxs) or cytotoxins (CTXs) are low-molecular-weight (6–9 kDa) non-enzymatic toxins that comprise about 60–70% of elapid venoms [3,15]. These toxins are characterized by the presence of three distinct beta-sheet loops radiating from a hydrophobic disulfide core [16]. While some 3FTxs act as neurotoxins by blocking the nicotinic acetylcholine receptors, cytotoxins insert into the lipid bilayers of cellular membranes, leading to cell demise [17,18].

C-type lectins (CTLs/snaclecs) are predominantly found in venoms of viperids. Although structurally related to classical C-type lectins, most snaclecs lack carbohydrate binding activity [19]. They specifically target different components of the hemostatic system. They interact with platelet receptors, coagulation factors and endothelial components, thereby modulating platelet aggregation, thrombosis and vascular integrity [20]. Hence, depending on their targets, they may activate or inhibit platelet function, leading to thrombosis or hemorrhage during envenomation. The major snake venom toxin families, the cellular stress responses they induce and their downstream cellular consequences are summarized in Table 1.

## 3. Venom-Induced Cellular Stress Response

**Figure 1:**
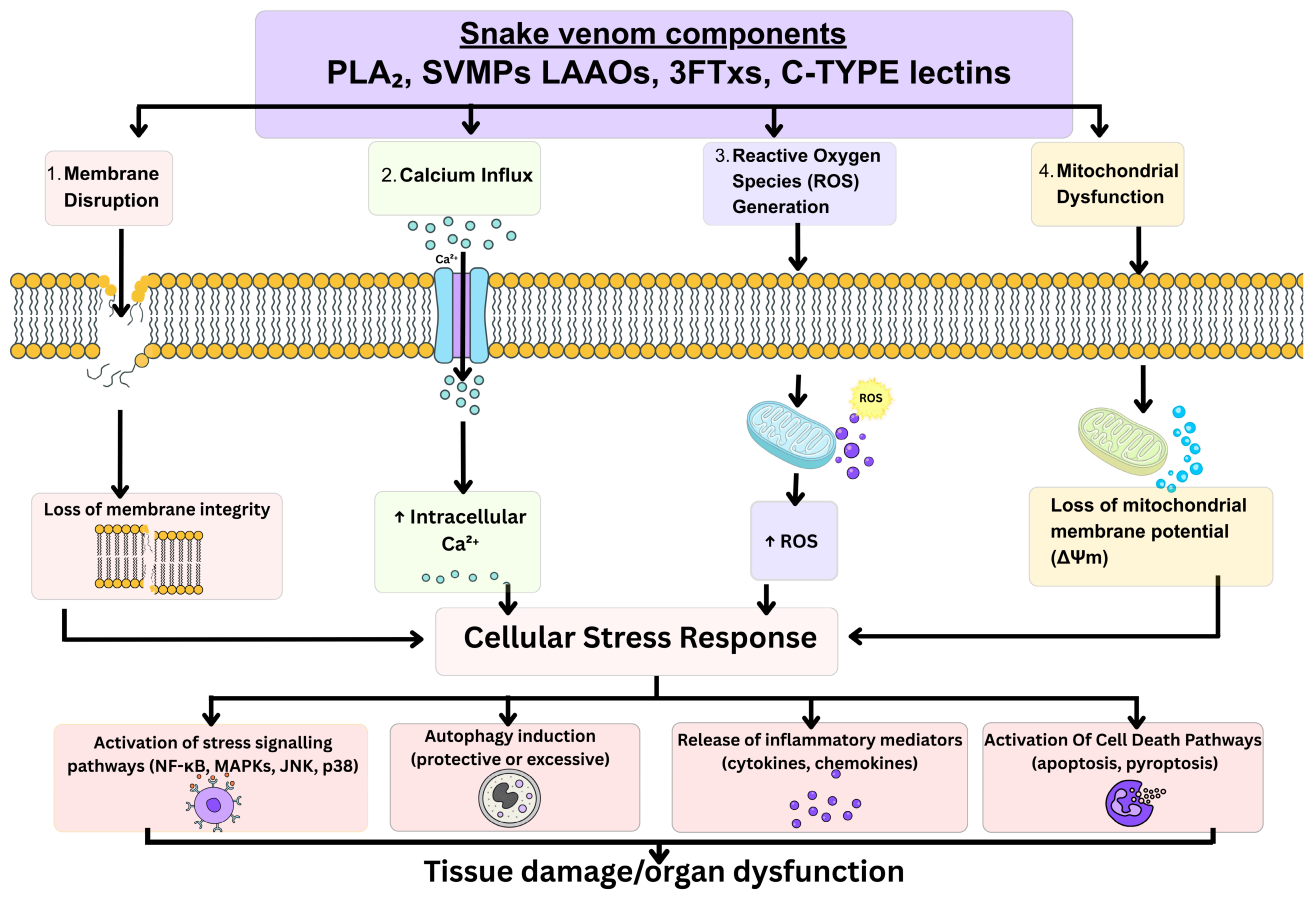
Snake venom-induced common cellular stress responses. Snake venom consists of diverse toxin families, including phospholipase A_2_ (PLA_2_), snake venom metalloproteinase (SVMP), L-amino acid oxidases (LAAOs), three-finger toxins (3FTxs) and C-type lectins (CTLs), which disrupt cellular homeostasis through multiple mechanisms. These toxins are implicated in membrane disruption, calcium influx, ROS production and mitochondrial dysfunction, leading to activation of cellular stress responses. This stimulates stress-related pathways (NF-*κ*B, MAPK, JNK, p38), autophagy, release of inflammatory mediators and activation of regulated cell death pathways. Collectively, these responses amplify local tissue damage and may contribute to systemic organ dysfunction following snakebite envenomation.

Local and systemic toxicity due to snakebite envenomation is primarily driven by cytotoxic venom components that affect cell viability and extracellular matrix degradation [4,26]. It has been observed that many venom components lead to a highly orchestrated cellular stress response (Figure 1) rather than bring about direct cytotoxic destruction. For instance, three-finger toxins and phospholipase A_2_ are the predominant venom components of cobra venom, along with metalloproteinases and L-amino acid oxidases (LAAOs). Apart from direct lysis, these toxins function as highly specific ligands that interact with host cellular targets, such as ion channels and membrane receptors [3]. This initiates a cascade of cellular stress, including oxidative imbalance and calcium dysregulation. These cellular responses then lead to a point of no return that transitions from sublethal cellular injury to a defined form of regulated cell death (RCD), such as apoptosis, ferroptosis, and pyroptosis [26]. Thus, inhibiting the activation of different RCD forms using adjunct therapies is crucial for mitigating the mortality and morbidity associated with snakebite envenomation. The main cellular stress responses induced by venom toxins include those shown in the figure below.

### 3.1. Oxidative Stress

The primary driver of venom-induced cytotoxicity is the disruption of cellular redox homeostasis. Both enzymatic and non-enzymatic venom toxins can contribute to oxidative stress through distinct mechanisms [8]. Enzymatic toxins such as LAAOs can directly generate H_2_O_2_ by oxidative deamination of amino acids, whereas PLA_2_ promotes ROS production indirectly by the disruption of membrane phospholipids, calcium overload and mitochondrial dysfunction. In contrast, non-enzymatic toxins like 3FTxs primarily induce ROS formation indirectly through membrane disruption, mitochondrial damage, calcium dysregulation and subsequent activation of inflammatory response. The resulting oxidative stress disrupts the cell’s antioxidant defense systems, leading to lipid peroxidation, protein modifications, and oxidative DNA damage, ultimately causing intracellular accumulation of ROS, which acts as the primary cause of pro-apoptotic and pro-necrotic signaling pathways.

### 3.2. Calcium Overload

Disruption of intracellular ion balance, particularly calcium ion imbalance, is a hallmark of venom-induced toxicity [26]. Venom toxins compromise cellular membrane permeability and alter certain ion channels, resulting in elevated intake of Ca^2+^ ions from extracellular spaces, along with the uncontrolled release of calcium stores from the ER. This intracellular calcium overload can activate calcium-dependent enzymes like caspases, calpains, and other hydrolases, which can degrade structural proteins and cytoskeletal components, leading to the execution of RCD pathways.

### 3.3. Mitochondrial Dysfunction

Mitochondria serve as a critical target that converts upstream cellular stress to downstream executioner death pathways. The combination of elevated ROS and calcium overload leads to a loss of mitochondrial membrane potential (MMP). This leads to a decrease in ATP synthesis and the release of cytochrome c, thereby leading to activation of RCD-like apoptosis [27,28].

### 3.4. Inflammation

Local tissue damage due to envenomation can be caused by a secondary sterile inflammatory pathway [29] that leads to the breakdown of cellular components, triggering the release of DAMPs into the microenvironment [30]. DAMPs then activate resident immune cells, triggering their binding to pattern recognition receptors, like Toll-like receptors (TLRs), thereby upregulating pro-inflammatory cytokines and promoting the inflammatory modes of RCDs, like pyroptosis.

### 3.5. Membrane Disruption

The initial biochemical assault induced by venom involves the disruption of cellular membranes by venom cytotoxins, thereby destabilizing the membrane integrity of cells. This is mediated by the enzymatic hydrolysis of membrane lipids or by the direct insertion of certain cytotoxins into the plasma membrane [2]. This can result in cellular swelling or the release of cellular contents, leading to severe necrosis or more regulated pathways for cellular destruction [19].

## 4. Overview of Regulated Cell Death Pathways

Regulated cell death refers to an active physiological process comprising genetically determined signal transduction pathways that function in the clearance of infected, neoplastic, or damaged cells [31,32]. Unlike accidental cell death (ACD), which results from overwhelming physical, chemical or mechanical injury that occurs in an uncontrolled manner, RCD is mediated by defined molecular signaling pathways. Hence, RCD can be modulated pharmacologically. Major forms of RCD include apoptosis, pyroptosis, ferroptosis, and autophagy, each with distinct molecular mechanisms and biological outcomes [33]. Besides eliminating damaged or infected cells, RCD plays a crucial role in tissue homeostasis, embryonic development, immune regulation and host defense. In the immune system, RCD regulates inflammatory responses, controls pathogen dissemination, facilitates antigen presentation and contributes to the resolution of inflammation. However, excessive activation of RCD can lead to pathological tissue injury and chronic inflammation [33]. ACD is characterized by rapid destruction of a cell and is generally considered uncontrollable [21,34] and pharmacologically non-modulatable. Several experimental studies have demonstrated the potential involvement of these RCD pathways in snake venom toxicity. However, evidence for some pathways is limited to in vitro and animal models.

Apoptosis is the most extensively studied form of programmed cell death, and unlike necrosis, it is immunologically silent [35]. It is mediated by the activation of caspases by two different pathways—the intrinsic and extrinsic (death receptor) pathways [36]. In contrast, pyroptosis is an inflammatory form of RCD mediated by activation of the inflammasome and caspase-1, resulting in membrane pore formation mediated by gasdermin and cytokine release [37,38]. Ferroptosis represents an iron-dependent RCD pathway characterized by lipid peroxidation and downregulation of the antioxidant defense of cells [39,40,41].

Autophagy is primarily a cellular survival mechanism when a cell is under stress. It involves the degradation of damaged cellular organelles, protein aggregates and other cytoplasmic components, mediated by the formation of an autophagosome and an autolysosome. Although autophagy is a survival mechanism, it can contribute to cell death when activated or dysregulated or modulate other RCD pathways through extensive crosstalk [42]. Therefore, in this review, autophagy is discussed as a cellular stress response pathway in the context of snakebite envenomation that influences apoptosis, pyroptosis and ferroptosis rather than a core regulated cell death.

## 5. Mechanistic Role of RCD in Snakebite Envenomation

### 5.1. Apoptosis

**Figure 2:**
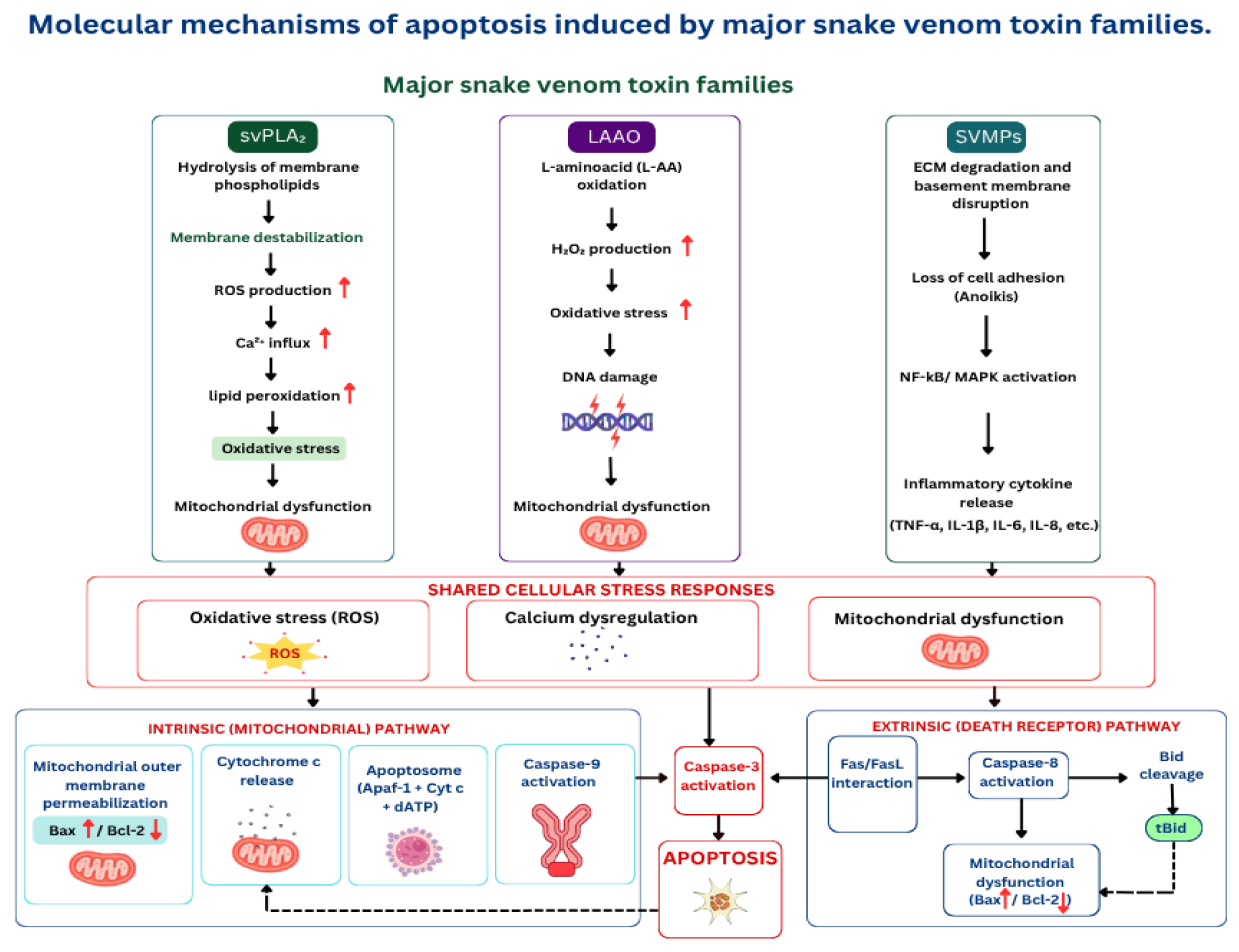
Molecular mechanisms underlying apoptosis induced by major snake venom toxin families. Major snake venom toxin families, including phospholipase A_2_ (PLA_2_), LAAO, 3FTxs and SVMPs, induce apoptosis through distinct and convergent mechanisms involving oxidative stress, calcium dysregulation and mitochondrial dysfunction. These cellular responses activate the intrinsic (mitochondrial) and/or extrinsic (death receptor) apoptotic pathways, leading to caspase-3 activation and apoptotic cell death.

Experimental studies suggest that venom PLA_2_ and LAAOs are the primary contributors to venom-induced apoptosis. In vitro evidence suggests that sv-PLA_2_s can promote ROS generation, which may elevate oxidative stress to disrupt cellular homeostasis [21]. This oxidative damage has been shown to correlate with the upregulation of pro-apoptotic proteins like BAD, the activation of caspase-3, and the downregulation of anti-apoptotic proteins like Bcl-2 and Bcl-XL (Figure 2) [43]. Snake venom L-amino acid oxidases catalyze the oxidative deamination of L-amino acids with the production of high levels of H_2_O_2_ [25]. Accumulation of H_2_O_2_ subsequently damages cellular membranes, leading to disruption of mitochondrial membrane potential [44]. This triggers the activation of both intrinsic (caspase-9-mediated) and extrinsic (caspase-8-mediated) apoptotic pathways, ultimately resulting in DNA fragmentation and cell cycle arrest [45,46].

Mojave A toxin (MTX) from the Mojave rattlesnake (*Crotalus scutulatus scutulatus)* is a highly potent presynaptic beta neurotoxin composed of both acidic and basic subunits of phospholipase A_2_. One study employed two concentrations of the pooled venom—10 and 30 µg/mL—to treat iPSC-derived neural stem cells. The cells were analyzed at two time points (4 h and 24 h) after treatment with the venom using genome-wide deep RNA sequencing. The genome-wide transcriptomic analysis of induced pluripotent stem cell (iPSC)-derived neural stem cells treated with Mojave A toxin showed that the venom can activate apoptotic pathways. The MTX PLA_2_ activity was shown to disrupt cell membrane integrity, leading to a rapid influx of intracellular calcium. This dysregulation of calcium homeostasis was shown to induce ER stress and upregulation of Unfolded Protein Response (UPR) in venom-treated NSCs [47]. Generally, UPR is activated by ER stress due to the accumulation of unfolded or misfolded proteins. This activated UPR tries to rescue cells from ER stress by two pathways—the PERK/EIF2AK3 pathway, which reduces protein synthesis, and the IRE1/ERN1 pathway, which increases protein folding [46]. However, unresolved ER stress due to venom exposure is proposed to lead to activation of the apoptotic pathway. Together, the experimental data suggests that Mojave venom-induced ER stress and oxidative stress lead to the activation of the integrated stress response, resulting in the phosphorylation of eIF2α. This reduces overall protein synthesis but upregulates other stress response genes, such as ATF and DDIT3/CHOP, and apoptotic genes, such as BAX, PMAIP1, and BBC3, resulting in mitochondrial membrane permeabilization and cytochrome c release [47]. Furthermore, the venom was also shown to upregulate death receptor genes, indicating the activation of the extrinsic pathway. Both pathways might converge at caspase-3, as indicated by the increased expression of the caspase-3 gene. Thus, the experimental data from this study indicates that Mojave toxin can activate the genes involved in both intrinsic (BAX, PMAIP1 (NOXA), and BBC3 (PUMA)) and extrinsic (TRAILR, TNFRSF10A, TNFRSF10B, and TNFRSF10D) apoptotic pathways in human iPSC-derived neural stem cells. However, there are several limitations in the study, which include correlative evidence that was not validated in a more complex biological system [47]. The study was mainly conducted in human iPSC-derived neural stem cells in an in vitro culture system. While these cells are relevant in vitro models for studying the neurocellular stress response, other cell types in a living organism might show a different result compared to that in an isolated cell culture system. Additionally, the study focused solely on transcriptional changes in venom-treated cells without experimental validation at the protein level or consideration of functional cellular consequences [47].

Another important observation suggests that snake venom PLA_2_ cytotoxicity by apoptosis in cells is not just dependent on its enzymatic activity but also induced by catalytically inactive PLA_2_s. Vipoxin is a heterodimeric neurotoxic complex isolated from the venom of *Vipera ammodytes* ssp. *Meridionalis*, which consists of a basic secreted, catalytically active PLA_2_ Vipoxin Basic Component (VBC) and an acidic non-catalytic subunit Vipoxin acidic component (VAC). Human retinal pigment epithelial cells (RPE-1 and ARPE-19) were exposed in vitro to vipoxin or its individual subunits: cytotoxicity was assessed at concentrations of 0.5–15 µM, DNA damage and apoptosis at 1–10 µM concentrations, and cytoskeletal rearrangements at 5–15 µM concentrations. Vipoxin basic component (VBC), when treated in retinal epithelial cells RPE-1 and ARPE-19, induced cytotoxicity and caused extensive DNA double-strand breaks (up to 60% damaged DNA), leading to apoptotic cell death in RPE cell lines. In contrast, VBC in ARPE-19 cells triggered necrotic cell death. VAC did not affect membrane integrity due to a lack of catalytic activity but induced DNA damage in ARPE-19 cells and triggered more necrotic cell death than apoptosis, whereas VAC in RPE-1 cells promoted apoptosis. Furthermore, both the vipoxin complex and its individual subunits—VBC and VAC—induced rapid and sustained activation of the p38-MAPK signaling pathway at a 5 µM concentration in RPE-1 cells and promoted cytoskeletal rearrangements, including actin filament reorganization in both cell lines. Thus, the vipoxin complex does not solely disrupt membrane integrity but also triggers DNA damage, cytoskeletal rearrangements, and activates the P38-MAPK pathway, impairing cell proliferation and leading to apoptotic cell death [21,48].

The experimental model used in the above study was human retinal pigment epithelial cells, and it was not validated in animal models. Hence, it is unknown whether the same mechanism occurs in vivo during snakebite envenomation. Moreover, the study was done in retinal cells and may not fully represent the local tissue damage occurring after envenomation, unlike the major target cell types at the bite site, like keratinocytes, fibroblasts, skeletal muscles, endothelial cells or macrophages. Furthermore, the study provided only limited evidence of apoptosis, based on Annexin V/PI staining and DNA double-strand breaks, and did not investigate the involvement of apoptotic machinery, namely, caspase-3, caspase-9, cytochrome c release, Bax/Bcl2 or PARP cleavage. Hence, apoptosis was not mechanistically established in the study. Additionally, the venom concentrations used were 5–15 µM, and it is unclear whether these concentrations are physiologically relevant after snakebite envenomation. As a result, the study demonstrated an association between vipoxin exposure and apoptotic signaling but failed to establish the precise molecular mechanism driving cell death in vivo [48].

LAAOs are present in both Viperidae and Elapidae venoms and are the primary mediators of oxidative stress-induced apoptosis. Batrox-LAAO and Cr-LAAO catalyze the oxidative deamination of amino acids, releasing H_2_O_2_. This H_2_O_2_ burst overrides cellular antioxidant defense systems, directly leading to DNA damage and inducing both intrinsic and extrinsic apoptosis involving caspase-9, caspase-8, and caspase-3 (Figure 2).

Snake venom metalloproteases (SVMPs) enzymatically degrade the structural components of the extracellular matrix and basement membranes, and this results in the detachment of cells from the basement membrane support, leading to apoptosis by a specialized mechanism called anoikis (Figure 2) [49,50,51]. This was shown when Jararhagin induced endothelial cell apoptosis in a time- and concentration-dependent manner by increasing DNA fragmentation and caspase-3 activation. The toxin disrupted the actin cytoskeleton, reduced FAK phosphorylation, and promoted detachment of cells from the extracellular matrix, thus inducing apoptosis via anoikis. These cytotoxic actions of jararhagin were selective to endothelial cells, as fibroblasts and peritoneal macrophages were resistant to this effect [49]. Another study revealed that exposing endothelial cells to atrase A, a P-III metalloprotease from cobra venom, induced an increase in ROS and a decrease in Ca^2+^ influx, mitochondrial membrane potential, and ATP levels. Additionally, the inflammatory reactions of the MAPK and NF-*κ*β signaling pathways were also shown to be upregulated by atrase A, leading to the release of inflammatory mediators (TNF-α, IL-6 and IL-8) [52]. This suggests that metalloproteases could induce tissue damage after a cobra bite.

### 5.2. The Inflammasome–Pyroptosis Axis in Snakebite Pathology

Snake envenomation can lead to host-driven inflammatory reactions coordinated by multimeric protein complexes known as the inflammasome. Specifically, the activation of NLRP3 (nucleotide-binding oligomerization domain, leucine-rich repeat, and pyrin domain-containing 3) inflammasome by snake venom toxins has been observed primarily in cell culture and animal models [53,54,55]. These inflammasomes within innate immune cells are understood to act as sensors for exogenous venom toxins and endogenous cellular danger signals, known as Damage-Associated Molecular Patterns (DAMPs), released by damaged cells during tissue injury. Activated by snake venoms, NLRP3 recruits the ASC adaptor protein and pro-caspase-1. This assembly activates caspase-1, leading to the release of the cytokines IL-1β and IL-18. Instead of leading to direct destruction of tissues through lytic degradation, the venom toxins integrate the host’s innate immune system, which leads to more inflammatory reactions that culminate in a highly regulated programmed cell death known as pyroptosis. Various enzymatic and non-enzymatic toxins in snake venom can activate the NLRP3 inflammasome.

#### 5.2.1. The Canonical Two-Step Inflammasome Activation Pathway

**Figure 3:**
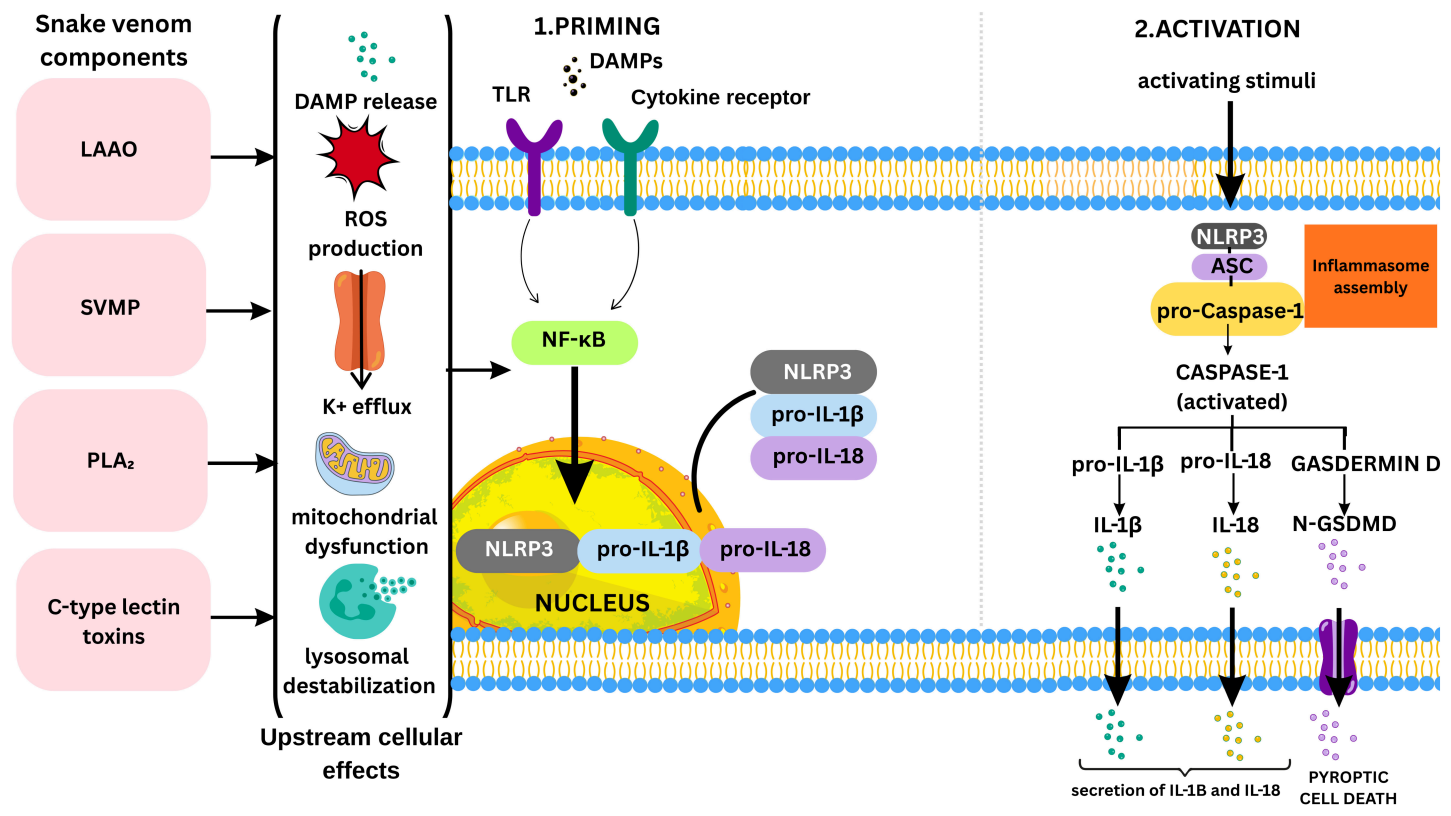
Molecular mechanisms of NLRP3 inflammasome-mediated pyroptosis induced by snake venom toxins. Major toxins like LAAO, SVMP, PLA_2_ and C-type lectins induce pyroptosis through a two-step process involving priming and activating the NLRP3 inflammasome. The upstream cellular stress signals activate NF-kB-dependent transcription of inflammasome components, followed by inflammasome assembly, caspase-1 activation, maturation of IL-1β and IL-18, gasdermin D cleavage and pyroptosis.

Several venom toxins like snake venom phospholipase A_2_ (PLA_2_), snake venom metalloproteinases (SVMPs), C-type lectins (CTLs), and L-amino acid oxidases (LAAOs) have been shown to activate the inflammasome through a two-step mechanism (Figure 3):

Signal 1—The priming phase:

When venom toxins are exposed to tissue, the lysed cells release endogenous DAMPs, like extracellular ATP and mitochondrial DNA, which bind to Toll-like receptors (TLR-2 and TLR-4) on host macrophages and neutrophils (Figure 2). For instance, an in vitro study using human peripheral blood mononuclear cells (PBMCs) demonstrated that the C-type lectin BjcuL from *B. jararacussu* directly engages TLR4, leading to the activation of the NF-kβ pathway and reprogramming the cell to activate the NLRP3 and pro-inflammatory cytokine genes IL-1β and IL-18. The release of IL-1B through TLR4 engagement was abolished when the cells were pre-treated with LPS-RS, which is a TLR4 antagonist. Additionally, they used molecular docking and molecular dynamics simulations to show that BjcuL forms a stable heterohexameric complex with the TLR4-MD2 receptor [55].

Phospholipase A_2_ (PLA_2_), which is the most abundant toxin in viperid venoms, has also been shown to activate NLRP3 in experimental setups. Using murine macrophages and human peripheral blood mononuclear cells (PBMCs), a study demonstrated that BthTX-1, a Lys49-PLA_2_ with non-catalytic activity and BthTX-II, an Asp49-PLA_2_ with catalytic activity from *Bothrops jararacussu,* activated the NLRP3 inflammasome, causing the release of IL-1β [53,56]. Both toxins were able to activate the inflammasome through the cyclooxygenase (COX) pathway, thereby leading to the assembly of the NLRP3 complex by the prostaglandin E2 (PGE2) signaling via the EP2 and EP4 receptors [56].

Signal 2—NLRP3 assembly and caspase-1 activation:

Following the priming phase, enormous intracellular perturbations lead to a secondary trigger where venom toxins induce elevated potassium (K+) efflux and mitochondrial dysfunction, along with increased production of reactive oxygen species (ROS) (Figure 2). In vitro treatment of human neutrophils with L-amino acid oxidases from *Calloselasma rhodostoma* (Cr-LAAO) demonstrated acute inflammation in human neutrophils. This was mainly shown by increased ROS production due to the activation of the NADPH oxidase complex and protein kinase C (PKC-α). This led to NLRP3 activation, as indicated by the accumulation of punctas in the cytosol. ROS production led to the oligomerization of the NLRP3 sensor, recruited the adaptor protein ASC and activated pro-caspase-1 to caspase-1. The study demonstrated that the NADPH oxidase-induced ROS formation was the main player in the activation of the inflammasome, as the protein expression of NLRP3, cleaved caspase-1, and IL-1β was significantly reduced by the treatment of the NADPH oxidase inhibitor apocynin [57].

#### 5.2.2. Execution of Canonical Gasdermin D-Mediated Pyroptosis

Activated caspase-1 acts as the primary effector protease and cleaves pro-IL-1β to mature IL-1β, as well as gasdermin D (GSDMD). Cleaved gasdermin D can oligomerize and insert into the plasma membrane of a host cell, forming membrane pores. This pore formation leads to lytic pyroptosis and release of IL-1β, LDH, and DAMPs to the extracellular space. Snake venom metalloproteases also contribute to tissue damage by activating the pyroptotic pathway. BjussuMP-II, a non-hemorrhagic SVMP from *Bothrops jararacussu,* can activate NLRP3 in human neutrophils. In one study, BjussuMP-II upregulated the NLRP3, ASC, and caspase-1 genes and the HIF-1a transcription factor, thereby leading to GSDMD-driven release of IL-1β. Thus, by upregulating HIF-a and GSDMD-dependent pore formation, BjussuMP-II may promote pyroptosis and trigger a cycle of sterile inflammation, leading to tissue destruction. However, the study has limitations since only three donors were utilized; hence, it is subject to interindividual variability. Additionally, the use of Western blots and dot blots may not be sufficient for detecting NLRP3 and degraded caspase-1 because of their low abundance [58]. Hence, these observations for venom-induced pyroptosis have been shown predominantly in experimental cell culture and animal models with purified venom toxins under controlled experimental conditions without any direct clinical validation.

#### 5.2.3. Pyroptosis-Independent Cytokine Release by Venom Toxins

Experimental evidence suggests that certain venom toxins can induce cytokine release, bypassing the pyroptotic pathway. For instance, the C-type lectin BjcuL and the L-amino acid oxidases Cr-LAAOs activate NLRP3 and caspase-1, leading to the release of IL-1β, but neither toxin leads to cleavage of GSDMD, showing that venom toxins can induce inflammatory cytokine release independent of the pyroptotic pathway, thus allowing immune cells to survive longer while inducing a cytokine storm [54,57]. However, these observations are limited to purified toxins, and whether pyroptosis-independent cytokine release occurs after whole-venom envenomation remains unclear.

#### 5.2.4. Cleavage of Alternative Gasdermins (GSDME)

Venom toxin can also induce cell death through cleavage of alternative gasdermin family members. In a murine skeletal muscle envenomation model, *Deinagkistrodon acutus* venom has been reported to bypass the canonical GSDMD-mediated pyroptotic pathway. Instead, the venom activates caspase-3, a key executioner caspase of apoptosis. Activated caspase-3 leads to the cleavage of gasdermin E (GSDME) and activation of pyroptosis, resulting in severe limb injury due to inflammatory muscle damage in skeletal muscles [59,60]. However, this mechanism has been shown only in the murine *D. acutus* envenomation model, and whether GSDME-mediated pyroptosis is conserved across other snake species or contributes to human snakebite pathology remains unknown.

#### 5.2.5. Non-Canonical Inflammasome Activation

Venom toxins can also induce the activation of non-canonical inflammatory pathways. For instance, an in vitro transcriptomic study using human thymic epithelial cells treated with *Bitis gabonica* venom reported a marked upregulation of the CASP5 gene, which encodes the inflammatory caspase-5. Additionally, there was also upregulation of NLRP1, CASP4, and CASP14 and downregulation of the CASP9 gene, which is the classical apoptosis initiator gene. These findings indicate that *Bitis gabonica* venom may cause thymic cell death through non-canonical NLRP1-dependent pyroptosis. This study highlights that envenomation from snakes like *Bitis gabonica* can severely impact the pathways within the immune system. The venom may induce pyroptosis in the thymus, which could cause a sudden decline in fresh naïve T-cells, leading to immune senescence. This may subsequently lead to a long-term risk to the victim’s health and immunity, leading to potential issues with autoimmunity and increased risk of cancer [60]. Although the study reported an unusually large increase in CASP5 expression that was independently evaluated and confirmed by conventional RT-qPCR, these findings are based only on gene expression analysis. Hence, further studies are required to demonstrate the activation of the NLRP1 inflammasome and pyroptosis at the protein and functional levels, as well as their relevance in animal models and human snakebite patients.

### 5.3. Ferroptosis

**Figure 4:**
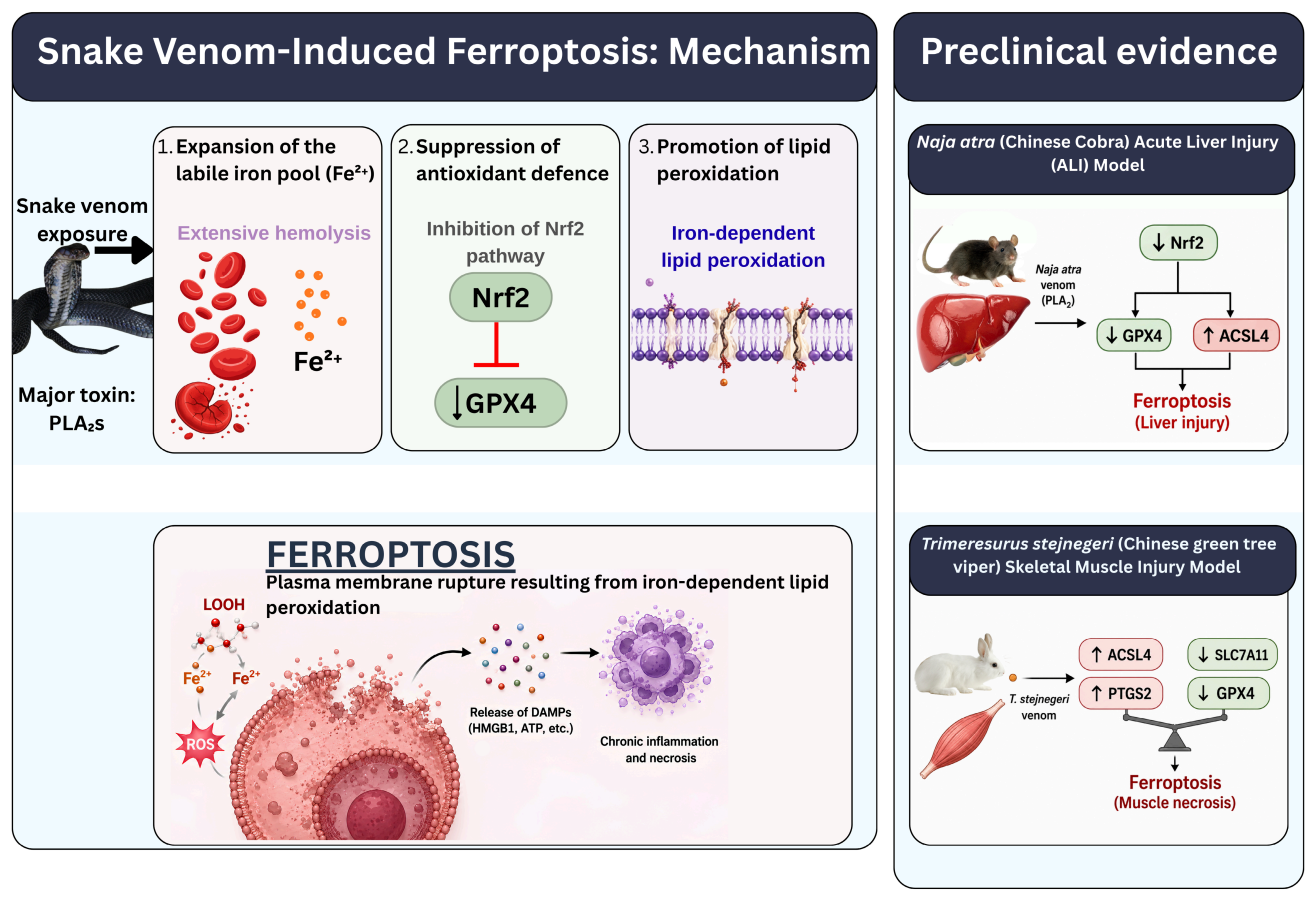
Molecular mechanisms and preclinical evidence of snake venom-induced ferroptosis. Snake venom promotes ferroptosis by dysregulating iron and redox homeostasis through expansion of the iron labile pool, suppression of the Nrf2-GPX4 antioxidant axis and iron-dependent lipid peroxidation, culminating in membrane damage and release of DAMPs. Representative preclinical studies in *Naja atra* and *Trimeresurus stejnegeri* demonstrate ferroptosis-associated molecular changes leading to liver injury and skeletal muscle necrosis, respectively. Adapted from [63,64].

Recent studies in clinical toxicology have identified ferroptosis as an iron-dependent, non-apoptotic form of regulated cell death characterized by uncontrolled lipid peroxidation, which serves as a key factor in the pathological descent following envenomation [61,62]. Snake venoms are protein pools containing high concentrations of phospholipase A_2_ (PLA_2_) and snake venom metalloproteinases (SVMPs) [62]. Experimental evidence suggests that these components induce a collapse of cellular redox homeostasis in several ways, such as expanding the labile iron pool through extensive hemolysis, suppressing the critical glutathione peroxidase 4 (GPX4) antioxidant axis, and promoting iron-dependent lipid peroxidation, which causes the plasma membrane to rupture and then release Damage-Associated Molecular Patterns (DAMPs), leading to chronic inflammation and necrosis [63]. A recent preclinical study using a mouse model explored acute liver injury (ALI) caused by *Naja atra* (Chinese Cobra) venom (Figure 4). The research demonstrated that venom PLA_2_ was associated with suppression of Nrf2, the primary regulator of the antioxidant response. This inhibition was shown to downregulate glutathione peroxidase 4 (GPX4) and upregulate the pro-ferroptotic enzyme ACSL4 (Acyl-CoA synthetase long-chain family member 4) [63]. Similarly, in a murine model of skeletal muscle injury induced by *Trimeresurus stejnegeri* (Chinese green tree viper), it was found that the venom upregulates pro-ferroptotic genes ACSL4 and PTGS2 and, at the same time, silences the protective genes SLC7A11 and GPX4 (Figure 4). It was observed that a traditional medicine against skeletal muscle necrosis triggered by the Chinese green tree viper brought back the cellular redox balance and acted as a broad-spectrum ferroptosis suppressor [64].

Despite these promising findings, evidence supporting the role of ferroptosis in snakebite envenomation remains limited. Current mechanistic findings are primarily derived from preclinical studies using murine models of *Naja atra* and *Trimeresurus stejnegeri* under defined experimental venom doses. Whether ferroptosis represents a conserved mechanism across other medically important snake species with distinct toxin compositions remains unclear. Moreover, direct evidence indicating the role of ferroptosis in human snakebite patients is currently lacking.

### 5.4. Autophagy and Mitophagy as Modulators of Venom-Induced Cell Injury

**Figure 5:**
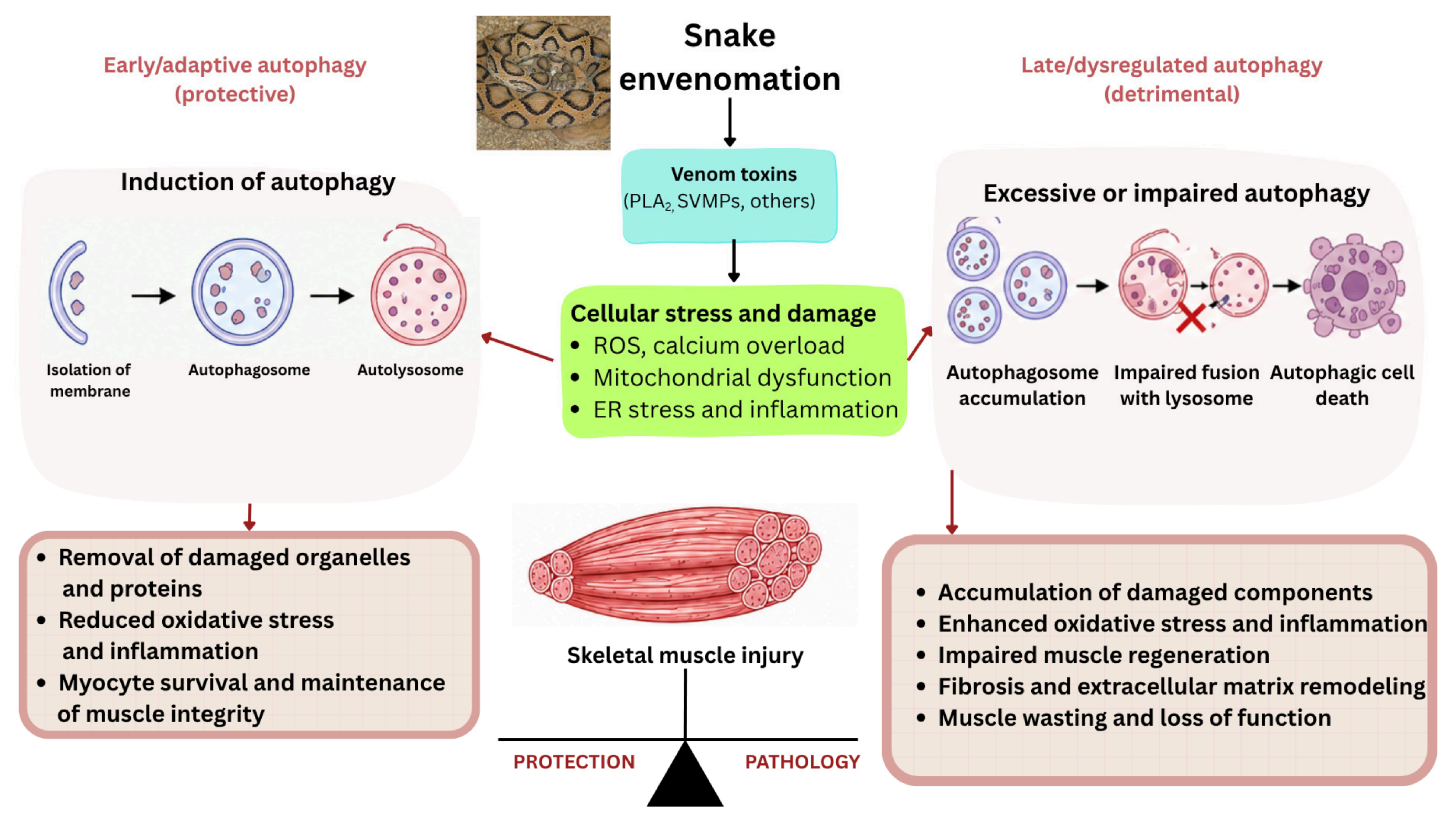
Protective and pathological roles of autophagy in snakebite envenomation. Snake venom toxins induce cellular stress through ROS production, calcium overload, mitochondrial dysfunction, ER stress and inflammation, leading to activation of autophagy. Early adaptive autophagy induces clearance of damaged organelles and proteins to maintain cellular homeostasis and muscle integrity. However, persistent venom exposure leads to impaired autophagy, characterized by autophagic flux and autophagosome accumulation. This increases oxidative stress, impairs muscle regeneration, and promotes fibrosis and ECM remodeling, ultimately contributing to skeletal muscle injury. (Image of *Daboia russelii* was adapted from Mike Prince, licensed under CC-BY-4.0).

According to the 2024 Nomenclature Committee on Cell Death (NCCD) recommendations, autophagy is not classified as a type of regulated cell death. Rather, it is a cellular process that can influence cell fate by promoting survival or modulating regulated cell death pathways. Autophagy is an intracellular degradation pathway that maintains cellular homeostasis by lysosomal recycling of damaged organelles, protein aggregates and cytoplasmic components. Thus, it serves as a protective mechanism but is pathogenic if dysregulated, contributing to atrophy and fibrosis (Figure 5) [65,66,67]. In the case of snakebite envenomation, autophagy may function as an adaptive response to clear venom-induced cellular damage, thus limiting uncontrolled necrosis. However, repeated venom exposure and cellular injury may dysregulate the autophagic process, contributing to tissue injury by interacting with other RCD pathways.

#### 5.4.1. Venom-Induced Autophagosome Formation

Currently, there is limited evidence that links snake venom exposure to autophagy, with only a few experimental studies reported to date. The best characterized example involves the activation of a rapid autophagic response when normal human keratinocytes were exposed to a purified L-amino acid oxidase (LAAO) from *Bothrops atrox* venom. Within 6 h of exposure to the venom, the LAAO induced cytotoxicity with an EC_50_ of 5.1 µg/mL, and mechanistic studies were performed at 10.2 µg/mL. This was shown to induce an early autophagic response within 6 h, characterized by increased autophagosome formation. However, continued venom exposure, oxidative stress and mitochondrial dysfunction overwhelmed this protective response, resulting in the activation of apoptosis at 12 h, followed by progressive necrosis between 12 and 24 h. These findings indicate that autophagy serves as a cytoprotective adaptive response rather than a regulated cell death pathway; however, irreversible cell death occurs when cellular injury exceeds the capacity of the autophagic process [68].

#### 5.4.2. Venom-Induced Mitophagy

Mitophagy is a form of autophagy where a cell removes damaged or dysfunctional mitochondria due to ROS accumulation or inflammatory reactions. The cell will exclusively remove damaged mitochondria to prevent further tissue destruction. While in the basal form, mitophagy plays a protective role, but excessive triggering can result in over-activation of mitophagy and damage to healthy mitochondria, ultimately damaging the organ [69,70,71]. An in vivo rodent model indicates that *Naja atra* venom induces severe liver injury due to excessive activation of mitophagy, as observed by increased elevation of the mitophagy biomarkers PINK1 (PTEN-induced kinase 1) and Parkin [63]. Under normal conditions, PINK1 enters mitochondria and gets degraded. When mitochondria are damaged, the mitochondrial membrane potential decreases, and PINK1 cannot enter, thus accumulating on the outer mitochondrial membrane. This activates Parkin, triggering its E3 ubiquitin ligase activity and ubiquitinating outer mitochondrial membrane proteins [72]. These proteins are then recognized by LC3 for ubiquitin-mediated degradation of damaged mitochondria. The study identifies sv-PLA_2_ as the central mediator of this destruction, as treatment with the PLA_2_ inhibitor varespladib was able to reverse the excessive mitophagy, thereby restoring mitochondrial homeostasis [63].

## 6. Crosstalk Between RCD Pathways in Snakebite Envenomation

Envenomation does not cause an isolated cellular event but a complex interconnected system where one toxic event triggers a cascade of multiple events and different forms of cell death sequentially or simultaneously. Recent multi-omics and molecular studies with different venom toxins show that the different RCD pathways, including apoptosis, ferroptosis, and pyroptosis, are deeply interconnected. The shared molecular nodes of different pathways are disrupted by different venom toxins, resulting in a complex crosstalk between these pathways, where failure of one adaptive response activates another pathway (Table 2).

Among the common cellular stress responses, oxidative stress (ROS production) represents one of the major molecular nodes that link different RCDs during envenomation. For instance, snake venom PLA_2_ hydrolyses membrane phospholipids and releases arachidonic acid. As a result, it can generate enhanced ROS production, which activates several RCD pathways. This was revealed by the transcriptomic analysis of svPLA_2_ Mojave Type A rattlesnake venom-induced pluripotent stem cell (iPSC)-derived neural stem cells, which gave insights into the activation of ferroptosis. The study revealed that there was an enhanced release of arachidonic acid due to the hydrolysis of the plasma membrane. Specifically, PLA_2_-mediated arachidonic acid metabolism by the cellular cytochrome P450 enzyme CYP1A1 promoted oxidative stress, resulting in lipid peroxidation and accumulation of iron, which are the molecular hallmarks of ferroptosis [34].

Venom toxin-mediated disruption of intracellular calcium homeostasis results in direct crosstalk between cellular stress adaptation and apoptosis. As previously mentioned, loss of membrane integrity by Mojave Toxin (MTX) leads to a rapid influx of calcium into the cytosol. Simultaneously, this depletes ER calcium stores, leading to ER stress and activation of UPR mediated by stress kinases to decrease protein synthesis and restore cellular homeostasis. However, when ER stress remains unresolved due to a high concentration of venom, transcription factors such as ATF4 and DDIT3 (CHOP) are upregulated by UPR kinases. This will transition the cell fate from adaptation to destruction by transcribing apoptotic genes, resulting in intrinsic and extrinsic apoptosis [47].

Crosstalk between different pathways mediated by venom toxins has been observed in many studies. For instance, the Nrf2 (nuclear factor erythroid 2-related factor 2) signaling pathway can connect ferroptosis and apoptosis when hepatocytes are exposed to *Naja atra* venom. The transcription factor Nrf2 acts as a central regulator of the host’s primary antioxidant defense system [73,74] and determines cellular fate during envenomation. Snake venom PLA_2_ suppresses the Nrf2 protein, and since Nrf2 is involved in the normal regulation of lipid peroxidation, this suppression leads to downregulation of the antioxidant enzyme glutathione peroxidase 4 (GPX4) and upregulation of the pro-ferroptotic enzyme ACSL4 (Acyl-CoA synthetase long-chain family member 4). Subsequently, it leads to iron-dependent ferroptotic cell death in hepatocytes [63]. In addition to promoting ferroptosis, suppression of Nrf2 enhances oxidative stress and mitochondrial dysfunction, thereby lowering the threshold for apoptosis. Therefore, these findings suggest that Nrf2 functions as a common upstream regulator connecting multiple regulated cell death pathways rather than activating independently within the ferroptotic pathway.

Apart from ferroptosis, the suppression of Nrf2 can also lead to a transition to mitophagy and apoptosis. There is a marked accumulation of ROS simultaneously when Nrf2 is suppressed, thereby impairing the mitochondrial membrane potential. The mitochondrial damage then triggers the activation of PINK1 and Parkin, resulting in enhanced mitophagy. When mitophagy fails to remove damaged mitochondria, it leads to the release of cytochrome c into the cytosol, activating the pro-apoptotic enzymes caspase-3 and caspase-9. Thus, Nrf2 suppression is the common trigger that links ferroptosis and apoptosis, while mitophagy serves as an adaptive cellular stress response that modulates this process [63].

Similarly, Cr-LAAO is an L-amino acid oxidase isolated from the venom of the Malaysian pit viper *Calloselasma rhodostoma*. A recent study demonstrated that Cr-LAAO, through the production of H_2_O_2_ during oxidative deamination of amino acids, induces ROS formation, which activates the NADPH oxidase complex in neutrophils. This leads to increased superoxide generation through respiratory burst, further enhancing oxidative stress. This venom-induced release of ROS served as signal 2 for the inflammasome activation in human neutrophils. The elevated levels of ROS accumulation led to the oligomerization of the NLRP3 inflammasome, activating caspase-1. Activated caspase-1 then resulted in the degradation of gasdermin D, whose N-terminal fragment oligomerized within the plasma membrane to form pores. This led to cell swelling, membrane rupture and release of DAMPs, further activating inflammatory signaling, including inflammasomes. Therefore, these findings indicate that venom-induced oxidative stress also functions as a common upstream mediator of pyroptosis. This highlights ROS as a shared molecular node that may facilitate crosstalk with other regulated cell death pathways in the context of snakebite envenomation [57].

Autophagy was the earliest initial cellular stress response observed when normal human keratinocytes were exposed to *Bothrops atrox* and *Lachesis muta muta* snake venom [75]. In a study using *L. muta muta*, after 6 h, cells formed autophagosomes as an early adaptation to clear the toxic stimuli and damaged organelles. However, after 12 to 24 h, autophagy was suppressed due to venom-induced oxidative stress, and the cells transitioned into apoptosis and secondary necrosis. These findings suggest that autophagy is activated initially as an adaptive response to remove damaged organelles due to venom exposure and maintain cellular homeostasis before irreversible cell death pathways become predominant. Hence, venom-induced cell death is a chronological event rather than an instantaneous lytic event [68].

Recent studies published during 2024–2025 have considerably expanded our understanding of venom-induced host responses beyond the classical apoptosis pathway. Collectively, these studies suggest that oxidative stress, membrane disruption, calcium dysregulation, mitochondrial dysfunction, inflammatory signaling and disturbances in iron homeostasis function as interconnected upstream events that integrate apoptosis, pyroptosis, ferroptosis and the modulatory effects of autophagy. Rather than representing independent mechanisms, these pathways likely operate as a coordinated cellular stress response that ultimately determines the severity of venom-induced tissue injury. However, temporal sequences and tissue-specific interactions among these pathways remain poorly understood and warrant further investigation.

Another challenge in understanding venom-induced activation of RCD is the apparent tissue-specific activation of different cell death pathways. As previously mentioned, ferroptosis has been predominantly reported in hepatocytes following exposure to PLA_2_ from *Naja atra,* whereas GSDME-dependent pyroptosis has been reported in skeletal muscle following *D. acutus* envenomation. Similarly, transcriptomic studies of *Bitis gabonica* venom have demonstrated non-canonical pyroptotic signaling in thymic epithelial cells. However, it remains unclear whether these differences reflect tissue-specific susceptibility, differences in toxin distribution and concentration, differences in antioxidant capacities, iron metabolism or experimental models employed. Therefore, direct comparisons between tissues under standardized experimental conditions are lacking. Hence, future studies should investigate molecular mechanisms that result in tissue-specific activation of RCD pathways during snakebite envenomation.

**Table 2:** Summary of molecular crosstalk: shared signaling nodes and interconnected RCD pathways activated by specific snake venom toxins.

Venom Toxin	Toxin Family	Activated RCD Pathway	Shared Molecular Nodes	Refs
Mojave Toxin (MTX)	svPLA_2_	Apoptosis	Calcium influx, ER stress (UPR), ATF4, CHOP	[47]
Vipoxin basic component (VBC)	svPLA_2_ (active)	Apoptosis	DNA double-strand breaks, p38-MAPK pathway	[48]
Vipoxin acidic component (VAC)	svPLA_2_ (non- catalytic)	Apoptosis	DNA damage	[48]
Jararhagin	SVMP (PIII)	Apoptosis (anoikis)	Actin cytoskeleton disruption, reduced FAK phosphorylation, DNA fragmentation	[49]
atrase A	SVMP (PIII)	Apoptosis	ROS, decrease in mitochondrial membrane potential, NF-kB, MAPK	[52]
BjussuMP-II	SVMP (non- hemorrhagic)	Pyroptosis	HIF-1a transcription factor, GSDMD pore formation	[58]
Batrox-LAAO/ Cr-LAAO	LAAO	Apoptosis, pyroptosis	H_2_O_2_ burst, ROS, NADPH oxidase, PKC-a	[57,68]
BjcuL	C-type lectin (CTL)	Pyroptosis	TLR4 engagement, NF-kB activation, NLRP3 activation, cytokine release	[54]
BthTX-1/ BthTX-II	PLA_2_ (Lys49/ Asp49)	Pyroptosis	COX pathway, prostaglandin E2 (PGE2)	[56,63]
*Naja atra* venom	Whole venom	Ferroptosis, apoptosis	NRf2 suppression, GPX4 downregulation, ACSL4 upregulation, ROS	[63]
*Deinagikistrodon* *acutus* venom	Whole venom	Pyroptosis (GSDME- dependent)	Caspase-3 activation, GSDME cleavage	[76]
*Bitis gabonica* venom	Whole venom	Pyroptosis	Caspase-5, NLRP1 upregulation	[60]

## 7. Therapeutic Strategies

While the only approved treatment currently relies on intravenous administration of antibody-based antivenoms, which neutralize free toxins in systemic circulation, their large molecular size severely impedes tissue penetration, making them ineffective in reducing rapid local tissue damage [77]. In addition to poor tissue penetration of antivenom, geographical and interspecies variation in venom composition may further influence the neutralization of tissue-damaging toxins and contribute to variability in clinical outcomes. Additionally, antivenom is not available in all healthcare facilities, particularly in rural and resource-limited regions where snakebite incidence is highest. This delay in administering antivenom may reduce its effectiveness, as venom toxins can rapidly initiate irreversible pathological processes before the treatment is delivered. As a result, this delayed treatment is often associated with poor outcomes and an increased risk of permanent tissue damage [78]. Furthermore, substantial geographical [79,80], ontogenic [81] and interspecies and intraspecies variation in venom composition can influence the activity of tissue-damaging toxins, resulting in variable neutralization by conventional antivenom [82]. Hence, there is an urgent need to develop adjunct small-molecule and biological therapeutics that complement antivenom by modulating venom-induced inflammatory responses and regulated cell death pathways that may contribute to local tissue damage, thereby reducing morbidity and mortality.

Based on these considerations, several adjunct therapeutic strategies that target different stages of snakebite pathology have shown promising preclinical efficacy, including direct venom toxin inhibition, modulation of host inflammatory and regulated cell death pathways and next-generation biological therapeutics with improved toxin neutralization abilities.

The therapeutic strategies discussed in this section are predominantly supported by in vitro or preclinical animal studies and therefore should be considered investigational adjunctive approaches rather than clinically validated treatments.

### 7.1. Small-Molecule Inhibitors

Varespladib (LY315920) is a repurposed inhibitor of snake venom PLA_2_ [83,84,85,86] and is one of the most advanced adjunct therapeutics under investigation. In preclinical studies, varespladib has shown broad-spectrum protection against venom-induced toxicity from diverse snake species, including attenuation of myonecrosis, coagulopathy, neurotoxicity and tissue damage. Additionally, clinical trials (Phase II clinical trials) have demonstrated that oral varespladib methyl is safe and well tolerated as an adjunct to antivenom, supporting its potential for early intervention in snakebite management [87]. Recently, varespladib has been shown to rescue host cells from multiple RCD pathways apart from mere inhibition of enzymatic lipid hydrolysis mediated by the venom toxin. As previously mentioned, a preclinical *Naja atra* envenomation model demonstrated that varespladib effectively mitigated svPLA_2_-mediated suppression of Nrf2 signaling, thereby preventing acute liver injury. Furthermore, varespladib was able to limit lipid peroxidation (ferroptosis), mitophagy, and intrinsic apoptosis by restoring the Nrf2 functionality [63].

#### 7.1.1. MCC950

Given the role of the NLRP3 inflammasome in venom-induced inflammation and pyroptosis, MCC950 has been investigated as a host-directed adjunct therapy to assess whether modulation of this pathway can attenuate tissue damage. MCC950 (CP-456773) is a selective small-molecule inhibitor of the NLRP3 inflammasome by preventing its oligomerization and assembly, thereby inhibiting caspase-1 activation and IL-1B maturation [88]. Increased activation of the NLRP3 inflammasome is a major contributor to venom-induced cytokine storm, and hence, the targeted inhibition of the multimeric inflammasome complex has shown significant tissue-protective effects. The small-molecule inhibitor MCC950 directly targets NLRP3 and prevents oligomerization of the inflammasome [89,90,91,92]. This has been shown to prevent the activation of caspase-1 and the subsequent increase in the release of IL-1β in preclinical models of *Naja naja* and Bothrops envenomation [92]. These findings provide preclinical evidence that modulating the NLRP3 inflammasome may attenuate venom-induced inflammation and pyroptosis.

#### 7.1.2. Disulfiram

FDA-approved disulfiram, a drug that was originally developed for alcoholism, has been recently repurposed as an inhibitor of pore-forming gasdermin D (GSDMD), the terminal executioner of canonical pyroptosis. Since GSDMD-mediated pore formation in venom-induced pyroptotic cell death has been implicated, inhibition of this pathway has been investigated as a host-directed adjunct therapeutic strategy. In an in vitro study, when human neutrophils were treated with Bothrops metalloproteinase, disulfiram could successfully suppress lytic pore formation, thereby reducing the extracellular release of DNA and inflammatory cytokines [58]. Collectively, these findings provide evidence that pyroptosis is an important target in venom-induced tissue injury and identify GSDMD inhibition as a potential host-directed adjunct therapeutic strategy.

#### 7.1.3. Cp40

The small-molecule inhibitor Cp40 is a cyclic peptide that targets the complement system by acting specifically as a C3 and C3b inhibitor. Snake venoms can activate the complement cascade and generate the anaphylatoxins C3a and C5a, which elevate the inflammatory response. Cp40 is a C3-targeted complement inhibitor that binds to C3 and its activated C3b fragment, preventing C3 activation and subsequent complement cascade activation. In an ex vivo human whole blood model, Cp40 has been shown to reduce upstream activation and suppress downstream inflammasome assembly and localized release of IL-8 and MCP-1 after *Bitis arietans* envenomation [93]. These findings show that although Cp40 does not directly target any regulated cell death pathways, complement inhibition can attenuate the amplification of venom-induced inflammatory reactions and associated tissue damage.

### 7.2. Next-Generation Biologicals: IMRC Exosome and Camelid Antibodies

#### 7.2.1. IMRC Exosome (IMRC-Exo)

Exosomes derived from Immune and Matrix Regulatory Cells (IMRCs) have the ability to suppress the alternative, GSDME-dependent pyroptotic pathway and have been demonstrated as a novel host-directed biological therapy. In a preclinical rabbit model of *Deinagikistrodon acutus* envenomation, localized administration of IMRC-exo was able to significantly reduce caspase-3 activation and GSDME cleavage, thereby protecting limb skeletal muscle from pyroptosis and severe inflammatory cell infiltration [76]. Although IMRC-exo was shown to effectively suppress GSDME-dependent pyroptosis, the precise molecular mechanism underlying this effect remains unknown. Current evidence indicates that the inhibition of GSDME activation is likely indirect through inhibition of upstream inflammatory signaling, including caspase-3 activation, rather than direct inhibition of GSDME.

#### 7.2.2. Camelid-Derived Heavy-Chain Antibodies (HcAbs)

Traditional antibody-based antivenom suffers from low tissue penetrability due to its relatively large size. However, camelids such as llamas and camels naturally have a special type of heavy-chain-only antibodies (HcAbs). Antibodies derived from these animals lack light chains, resulting in their reduced molecular weight. Hence, these antibodies allow for superior penetration into deeper, necrotic muscle tissues [94]. This was observed in a preclinical *Bothrops jararacussu* envenomation model, where administration of HcAbs remarkably suppressed venom-induced myonecrosis, reduced NLRP3 protein expression, and inhibited the localized release of IL-1β [95]. Hence, camelid-derived heavy-chain antibodies (HcAbs) represent a promising next-generation antivenom therapeutic strategy that has demonstrated preclinical efficacy against snake venom toxins.

Despite their promising therapeutic potential, several translational challenges remain. These camelid-derived heavy-chain antibodies may exhibit lower immunogenicity than conventional antivenoms due to their smaller size. However, their immunogenicity in humans remains to be established. Furthermore, their smaller molecular size may result in a smaller circulating half-life compared with conventional IgG-based antivenoms. Hence, further preclinical and clinical studies are required to optimize their pharmacokinetic properties.

## 8. Limitations of Current Studies

While studies investigating the role of RCD in snakebite envenomation can contribute to major insights into understanding the complexity of venom-induced pathophysiology, several limitations must be addressed in future research.

Most of the investigations on venom-induced activation of RCDs are derived from in vitro cell culture models such as keratinocytes, endothelial cells, and macrophages. These cell culture models fail to replicate the complex, multidimensional microenvironment of snakebite envenomation, involving vascular leakage, ischemia, and immune cell infiltration [96,97]. Moreover, there is considerable heterogeneity among experimental studies with respect to venom preparations (crude venom versus purified venom toxins), venom doses, cell types, animal models, and experimental endpoints. This heterogeneity makes direct comparison between different studies difficult and limits the reproducibility and translation of experimental findings.

Furthermore, most experimental studies have not evaluated potential sex- or age-related differences in venom-induced activation of regulated cell death pathways. Future studies should evaluate whether these biological variables influence the activation of RCD pathways and therapeutic responses following snakebite envenomation.

Although in vivo animal models provide valuable systemic insights into venom-induced pathology, they do not fully recapitulate the complexity observed in human snakebite envenomation [98]. To improve clinical relevance, researchers are now using human tissue-based models such as ex vivo human skin models [96]; however, these models also have limitations, as there is a complete lack of vascularization and an inability to mimic intravenous interventions, like the administration of antivenoms.

Another important limitation is the substantial geographical, ontogenic and interspecies variation in venom composition. Since different snake species contain different proportions of phospholipase A_2_, snake venom metalloproteinases, three-finger toxins and other toxin families, the predominant RCD pathways activated during envenomation may differ considerably. As a result, mechanistic findings derived from crude venom or purified toxins cannot be generalized across all medically important snake species.

Additionally, many studies have investigated purified toxins instead of whole venom. Whole venom is composed of diverse toxins that can act synergistically or antagonistically; therefore, the activation and crosstalk of regulated cell death pathways observed with purified or individual toxins may not accurately reflect the host response to whole venom.

The snake venom injection site is a highly heterogeneous microenvironment, and many effects depend on the concentrations of toxins. High concentrations of a toxin at the injection site rapidly overcome local cellular defenses [99,100,101], while lower concentrations of venom in adjacent tissues may trigger early adaptive responses and survival mechanisms like autophagy and UPR to manage cellular stress [101]. Moreover, most mechanistic studies have focused on local tissue injury at the bite site, whereas comparatively fewer studies have investigated systemic complications such as acute kidney injury, hepatotoxicity, cardiovascular dysfunction and coagulopathy. Toxin biodistributions, tissue susceptibilities and inflammatory responses differ between local tissues and distant organs; hence, the RCD pathways leading to local tissue destruction may not necessarily reflect those that contribute to systemic organ injury.

Another limitation is the lack of comparative studies evaluating adjunct therapies. Most inhibitors, including varespladib, MCC950, disulfiram, Cp40 and IMRC-exo, have been investigated independently using purified toxins or whole venoms from different venoms, experimental models, dosing regimens, and treatment windows, as well as for their outcomes. Hence, direct comparison of their relative efficacy is currently not possible. Moreover, the dose–response relationships and threshold levels of venom-induced cellular stress that determine the preferential transition between different regulated cell death pathways remain unknown. Therefore, standardized comparative studies are required to determine optimal therapeutic strategies and therapeutic windows for adjunct inhibitors.

The effectiveness of antivenom and adjunct therapies is highly dependent on the timing of administration. Local tissue destruction is initiated immediately after venom injection; hence, delayed treatment may not reverse tissue damage even after circulating venom toxins have been neutralized. Hence, adjunct therapies are likely to be beneficial when administered early alongside conventional antivenom. Additionally, venom variability may also influence the cellular stress response and regulated cell death pathways activated after snakebite envenomation. Therefore, therapeutic strategies targeting specific signaling pathways may not show uniform efficacy across all medically important snake species, highlighting the need for validation using geographically diverse venoms. Moreover, successful implementation of the adjunct therapies will also depend on their accessibility, affordability, stability and ease of administration, particularly in rural and resource-limited regions where the snakebite burden is greatest. Small-molecule inhibitors with good tissue penetration and favorable storage properties may offer practical advantages in resource-limited settings.

Additionally, it is challenging to distinguish the sequential overlap of different RCDs induced by venom toxins and to mimic the dynamic environment of a live human snakebite. Future studies should employ standardized experimental protocols, clinically relevant models, comparative investigations across medically important snake species and patient-derived samples to validate these mechanisms and facilitate the development of effective adjunct therapies.

## 9. Conclusions

Local tissue damage due to snakebite envenomation is not limited to direct cytotoxic effects of the toxins, but rather it activates different signaling pathways. Major venom toxins induce local tissue damage due to their synergistic action on host cellular components [96,102,103], leading to the activation of different RCDs. These signaling pathways are not isolated events, but they are interconnected via common molecular events like the accumulation of ROS and other cellular stresses.

Understanding of the crosstalk between various RCDs and signaling pathways is critical for overcoming the limitations of current treatments for snakebite envenomation. Current antivenoms, which are antibody-based, have limited efficacy in reversing local tissue damage due to their large size [104], which limits their penetration into deeper tissues or prevents RCD once they have been activated. Hence, future snakebite management should rely on a multitarget approach along with traditional antivenoms. Small-molecule inhibitors and highly penetrative biologicals could be developed to complement antivenoms. Ultimately, such integrated treatment strategies may be necessary to reduce local tissue damage, prevent permanent disability and improve the clinical outcomes of snakebite victims.

Based on the current evidence, future research should focus on identifying common upstream cellular stress responses, including oxidative stress, calcium dysregulation, mitochondrial dysfunction and inflammatory signaling. These mechanisms integrate multiple regulated cell death pathways and hence represent more attractive therapeutic targets than inhibition of downstream cell death pathways alone. Therefore, future research should prioritize integrated multi-omics approaches, temporal profiling of cell death activation, and validation in clinically relevant animal models and human tissues to identify biomarkers and facilitate adjunct therapies that can complement conventional antivenom.

## Figures and Tables

**Table 1:** Cellular stress responses and downstream consequences induced by major snake venom toxin families.

Toxin Type	Main Cellular Stress Response	Major Downstream Consequences	References
Phospholipase A_2_ (PLA_2_)	Membrane phospholipid hydrolysis, ROS generation, calcium overload, mitochondrial dysfunction, lipid peroxidation.	Apoptosis, ferroptosis, pyroptosis, autophagy and mitophagy.	[4,21]
Snake venom metalloproteases (SVMPs)	Degradation of extracellular matrix and basement membrane, cellular detachment, microvascular collapse, increased ROS generation.	Apoptosis, anoikis, pyroptosis.	[12,22,23,24,25]
L-amino acid oxidases (LAAOs)	H_2_O_2_ production, oxidative stress, mitochondrial and DNA damage.	Apoptosis, ferroptosis.	[12,25]
Cytotoxins/three-finger toxins (3FTxs)	Membrane disruption by direct insertion into lipid bilayer, increased calcium influx, lysosomal permeabilization, mitochondrial fragmentation.	Apoptosis, necroptosis-like death, pyroptosis.	[15,17]
C-type lectins	Endothelial dysfunction.	Pyroptosis.	[20]

## Data Availability

No new data were created or analyzed in this study. Data sharing is not applicable to this article.

## Abbreviations

The following abbreviations are used in this manuscript:

3FTxs	Three-finger toxins
ACD	Accidental cell death
ACSL4	Acyl-CoA synthetase long-chain family member 4
ALI	Acute liver injury
AMPK	AMP-Activated Protein Kinase
ASC	Apoptosis-associated Speck-like protein containing a CARD
COX	Cyclooxygenase
CTLs	C-type lectins
CTXs	Cytotoxins
CYP1A1	Cytochrome P450 family 1 subfamily A member 1
DAMPs	Damage-Associated Molecular Patterns
ECM	Extracellular matrix
ER	Endoplasmic reticulum
FAK	Focal Adhesion Kinase
GPX4	Glutathione peroxidase
GSDMD	Gasdermin D
GSDME	Gasdermin E
HcAbs	Heavy-chain antibodies
HIF-1α	Hypoxia-Induced Factor 1-alpha
IL-1β	Interleukin-1 beta
IL-6	Interleukin-6
IL-8	Interleukin-8
IL-18	Interleukin-18
IMRCs	Immune and Matrix Regulatory Cells
IMRC-Exos	Immune and Matrix Regulatory Cell Exosomes
iPSC	Induced pluripotent stem cell
LAAOs/svLAAOs	L-amino acid oxidases/snake venom L-amino acid oxidases
LC3	Microtubule-associated protein 1 Light Chain 3
LDH	Lactate Dehydrogenase
MAPK	Mitogen-Activated Protein Kinase
MCP-1	Monocyte Chemoattractant Protein-1
MMP	Mitochondrial membrane potential
MTX	Mojave toxin
NF-κB	Nuclear factor kappa-light-chain-enhancer of activated B cells
NLRP3	Nucleotide-binding oligomerization domain, leucine-rich repeat, and pyrin domain-containing 3
Nrf2	Nuclear factor erythroid 2-related factor 2
NSCs	Neural stem cells
PBMCs	Peripheral blood mononuclear cells
PGE2	Prostaglandin E2
PINK1	PTEN-induced kinase 1
PKC-α	Protein kinase C alpha
PLA_2_/svPLA_2_	Phospholipase A2/snake venom phospholipase A2
RCD	Regulated cell death
ROS	Reactive oxygen species
SVMPs	Snake venom metalloproteinases
TLRs	Toll-like receptors
TNF-α	Tumor Necrosis Factor-alpha
UPR	Unfolded Protein Response
VAC	Vipoxin acidic component (acidic non-catalytic subunit)
VBC	Vipoxin basic component (basic secreted, catalytically active PLA_2_)
